# Influence of post-partum BMI change on childhood obesity and energy intake

**DOI:** 10.1371/journal.pone.0224830

**Published:** 2019-12-12

**Authors:** Martha M. Téllez-Rojo, Belem Trejo-Valdivia, Elizabeth Roberts, Teresa Verenice Muñoz-Rocha, Luis F. Bautista-Arredondo, Karen E. Peterson, Alejandra Cantoral

**Affiliations:** 1 Center for Nutrition and Health Research, National Institute of Public Health, Cuernavaca, Morelos, Mexico; 2 Anthropology Department, University of Michigan, Ann Arbor, Michigan, United States of America; 3 Nutritional Sciences Department, School of Public Health, University of Michigan, Ann Arbor, Michigan, United States of America; 4 CONACYT, Center for Nutrition and Health Research, National Institute of Public Health, Cuernavaca, Morelos, Mexico; Universidade de Sao Paulo, BRAZIL

## Abstract

**Introduction:**

Association between parent’s Body Mass Index (BMI) and their children, has been widely documented. Individual, familiar and structural factors play a role in this relation. We analyzed the association between maternal BMI change during the first year post-partum and their offspring’s growth-trajectories and energy intake in their first five years of life.

**Objective:**

Compare growth-trajectories and children’s caloric intake according to post-partum mother´s BMI classification.

**Methods:**

The anthropometric assessment was taken in 935 mother-child pairs along the study period. Mothers were classified into four groups according to their BMI-trajectories in the post-partum. Children’s weight for height z-scores (WHZ) was compared among groups using random-effects regression models. A longitudinal comparison of children’s caloric intake by the maternal group was carried out.

**Results:**

At 42 months of age, infants from mothers that remained overweight during the first year post-partum had, on average, 0.61 SD higher WHZ than those from mothers who remained in a recommended BMI group (R-BMI) in the same period. At 60 months of age, children´s prevalence of obesity was almost twice in the maternal overweight group vs R-BMI group (14.2% and 7.3% respectively). Chances for a child of having an over caloric intake were 36.5% (95% IC: 6.6%, 74.8%) and significantly higher among children from overweight mothers than those from R-BMI mothers. The difference in children’s WHZ trajectory remained significant after adjusting for caloric intake, suggesting that contextual factors play a role in shaping children’s obesity. A concurrent ethnographic study with the study subjects provides suggestions as to what these factors might be, including changes in the food landscape.

**Conclusion:**

Children from overweight mothers tended to have a more caloric diet yielding a higher propensity to obesity. Contextual factors such as food landscape might contribute to childhood obesity beyond having an overweight mother. Pregnancy and post-partum is a window of opportunity for interventions to decrease the incidence of children’s overweight.

## Introduction

The prevalence of overweight and obesity (OWO) in children has increased worldwide [1]. Between 42.5 and 51.8 million children and adolescents (0–18 years) in Latin America are OWO, representing 20–25% of the total population in the region in this age range [2]. In Mexico, the prevalence of OWO has shown a constant and significant increase within the population in the 1988–2016 period, particularly in children, adolescents, and women. For children under five years of age (yoa), this prevalence increased from 26.7% to 33.5 from 1988 up to 2012 [3]. Currently, almost one of every 10 children presents overweight at the mentioned age. In schoolchildren (5–11 yoa) OWO increased from 25.8% to 32% from 1999 to 2012. [3]. The highest increase in this 28-years period has been in adolescent women, moving from one to almost four out of ten with OWO (11.1% to 38.5%) and from 34.5% to 75.6% of women of reproductive age (20–49 yoa)[4,5], making Mexico one of the countries with the largest prevalence of OWO worldwide, and with a rapid increase [6].

Obesity is the result of complex factors that interact affecting individuals at different levels in different stages of life. As Rivera et al., [2] argue based on their conceptual framework for obesity, the *immediate causes* are the positive balance of energy mediated by physical activity, genetic and epigenetic factors. However, these *immediate causes* are influenced by *underlying causes* such as food access (processed or not), food costs, eating habits within families, and accessibility to environments that promote physical activity. These *underlying causes* are in turn, influenced by *basic causes* such as changes in family structure, urbanization and globalization [5].

Parental obesity is recognized as one of the most significant *immediate predictors* of childhood obesity [7,8] through a mix of genetic, epigenetic, and behavioral influences [9–11]. Additionally, an over-caloric intrauterine environment is positively associated with obesity later in life [12]. However, the rapid worldwide increase in obesity in the last three decades, and particularly in Mexico, suggests that environmental and structural factors play an important role in this phenomenon [5]. In the first years of life, family habits deeply influence children’s diet and physical activity. However, family decisions are influenced by *underlying* and *basic* causes, such as built environments and food landscapes that promote or inhibit physical activity and healthy diets [13].

The association between the parent’s BMI and their children has been widely documented [14,15]. The strongest association has been found when both parents are OWO [16–18] and at the upper end of the BMI distribution, with no major intergenerational changes when parents have a recommended BMI< 25kg/m^2^ [19]. Evidence on the differential influence of the father and mother is heterogeneous. Several studies have reported similar associations between the paternal and maternal BMI, and that of their children [18,20–24], however, some literature indicates that mothers have a greater influence on their children´s BMI [15,25–28], particularly, in early life [29]. The majority of the mentioned studies have been carried out in cohorts from developed countries and only a few on Hispanic populations [30,31] living in the US, so there is a lack of longitudinal studies in developing countries.

The objective of this study was to compare growth trajectories and to estimate the prevalence of overweight (>2SD WHZ score) in children in their first five years of life according to mother´s BMI classification along one year post-partum; and also, to conduct a longitudinal comparison of children’s caloric intake according to the above mentioned maternal group-classification. We hypothesized that those children whose mothers became or stayed obese during post-partum have hypercaloric diets and more chances of presenting overweight compared to those children whose mothers stayed in the recommended BMI.

## Materials and methods

### Study design and population

We studied mother–child pairs from the *Early Life Exposure in Mexico to Environmental Toxicants* (ELEMENT) project. The sample population pooled participants of four successively birth cohort studies, recruited between 1994 and 2004 (the same period when the rising of overweight in children started) at 6 public maternity hospitals, which serve low to moderate income populations in Mexico City.

The four cohort studies had their specific aims, but shared eligibility and exclusion criteria allowing estimation of pooled effects. Anthropometrical measurements and relevant covariates were collected with a standardized technique by the same group of investigators and fieldwork staff.

Cohort 1 [32] (n = 617) [33–35] and cohort 3 (n = 393) were randomized control trials to assess the effect of maternal calcium supplementation on blood lead levels and both, followed the mothers for one year post-partum. Cohort 2 (n = 686) is comprised of two observational studies; only the subcohort that was designed to follow the mothers for one year after delivery was included in this analysis (n = 253) [36]. More details about the running of each cohort have been published elsewhere [37,38].

Depending on the design of the specific cohort study, mothers were interviewed between two and four times during the first year post-partum. Children were assessed every six months in their first four (cohort 1) or five (cohorts 2 and 3) years of life. Women were not eligible to participate in the cohorts if they were planning to change residency from Mexico City within the following five years after recruitment; had a history of infertility, diabetes, or psychosis; daily consumption of alcohol during pregnancy; addiction to illegal drugs; physician’s diagnosis of current multiple or high-risk pregnancy; or did not intend to breastfeed (as one of the aims of the original cohort studies was to assess pregnancy and lactation as stimuli to mobilize bone lead into the bloodstream). Eligibility criteria to be included in this study were mothers with at least two anthropometrical measurements in the first year post-partum and children with at least one anthropometric measurement during infancy. Among the 1263 mother-child pairs that fulfilled these criteria, 935 were included in this study based on the availability of information for the analysis.

All research protocols were approved by the Research, Ethics and Biosafety committees of the National Institute of Public Health of Mexico and the Internal Review Board of the Harvard School of Public Health, the Brigham and Women’s Hospital, the University of Michigan School of Public Health, and the participating hospitals. All data were fully anonymized before you accessed them and the patients provided informed written consent to have data from their medical records used in research.

### Data collections and measures

We analyzed the intra-family variation of the feeding decisions process through the maternal BMI trajectory given that within most Mexican households, mothers, rather than fathers, are responsible for feeding their children [39,40]. Maternal height was measured once, using professional scales (BAME Mod 420) read to the nearest 0.5 centimeter (cm) and maternal weight was recorded once to the nearest 0.1 kilogram (kg). BMI was calculated as weight divided by height squared (kg/m^2^) and then classified following WHO guidelines [41]: Recommended: 18<BMI≤25; Overweight: 25<BMI≤30; and Obese: BMI>30. Children’s anthropometry (weight, height) was collected by standardized study personnel using established research protocols [42] calibrated beam scales (model TD16; Oken, Naucalpan, México). Weight was measured to the nearest 100gr and height was measured to the nearest 0.1 cm. We used these measurements to calculate WHZ based on WHO standards as the recommended indicator in children <5 yoa using the *WHO Anthro* macros [43].

Trained personnel administered a semi quantitative food frequency questionnaire (FFQ) to the mothers at each study visit to inquire on children’s diet over the past 3 months, beginning when the child was 12 months old. This instrument was validated in Mexican population using the Willet methodology [44]. Caloric intake was estimated using a nutritional composition database of foods compiled by the National Institute of Public Health [45]. Following the methodology proposed by Flores et al.[46], we identified all caloric intake below 500 or above 2500 as extreme outliers and were excluded from these analyses.

Status and duration of breastfeeding were determined by questionnaires administered to the mothers in each study visit. Maternal education, measured as years in school, was used as a proxy for socioeconomic status (SES) since ELEMENT did not collect information on SES in Cohort 1. Gestational age (GA) was calculated based on the date of last menstruation and the date of birth of the child, we consider a child as a premature for GA less than 37 weeks. Parity was defined in two group, as primiparous and multiparous. We classified marital status as with/without a partner. Overweight children was defined as >2SD of the WHZ score.

### Statistical analysis

We performed a data exploratory analysis to describe the distributional behavior of the main variables as well as to identify the association patterns among them. This included cross-sectional and longitudinal analyses of the information aimed to establish the kind of children growth curve, types of maternal changes in BMI, and relationships between them and relevant covariates.

According to BMI changes along the study period, women were classified in four groups that we called *Post-partum BMI pattern (PP-BMIP)*: 1) mothers who stayed within the recommended BMI(R-BMI); 2) mothers who stayed within OWO categories; 3) mothers who moved from OWO to R-BMI and 4) mothers who moved from the R-BMI to OWO.

We used maternal anthropometry in the first year post-partum as an indirect way to assess pregnancy weight as many studies has done before [47–50], where gestational weight gain explained almost the majority of the variability in post-partum weight change[51]; as well as family and social environments under the hypothesis that mothers who remained OWO tend to feed their children differently with respect to mothers that remained in the R-BMI. To better understand these relationships, we undertook a statistical approach in two stages: 1) comparisons of children’s caloric intake between *Post-partum BMI pattern*; 2) modeling weight for age z-scores trajectories during the study period according to these groups.

Cross-sectional analysis of the caloric intake per study visit suggested the existence of two types of infant’s diet overall: *recommended caloric intake* and *high caloric intake*. This was confirmed by the adjustment a 2-normal mixture model [52]. We carried out a longitudinal analysis to compare the *children diet groups* along the study period according to *Post-partum BMI pattern* based on a logistic model with random effects adjusted by sex and child´s age.

Kernel density estimators for WHZ along every study visit by these maternal groups were obtained showing a symmetric and unimodal distribution in any case. Modeling WHZ along the study period according to *Post-partum BMI pattern* was assessed using a linear regression model with random effects [53] adjusted *by children diet group*, birth weight, duration of breastfeeding, maternal age, and maternal education. To account for the rapid increase in prevalence of OWO in Mexico, models were adjusted by year of birth. The quadratic relationships between maternal education and year of birth with the outcome were modeled using a second degree polynomial for each year. All statistical analyses were conducted in STATA 14 (StataCorp LP, College Station, TX, USA).

To understand the variability that was not explained by the statistical analysis, we used results from an ethnographic study [44, 45] conducted from 2013–2016 in a subset of six families of our study population which aimed to understand daily eating and physical activity habits within the social, economic and political environments through bi weekly intensive observations of family daily routines including neighborhood activities. [46–50] These observations were documented through field notes, which were subsequently coded in the qualitative coding program, Atlas Ti. The coding focused on patterns of everyday life especially in regards to the purchasing, preparing, serving, sharing and consuming food within families and within neighborhoods. Because of their primary role in food preparation, ethnographic observations centered on mothers as their fed their families.

## Results

### Baseline characteristics

Mother’s age ranged from 14 to 44 years at one month post-partum with a mean of 26.21(5.4). On average, women attended school 10.39(3.0) years; most of them (90.56%) had a partner and for 26.7% this was the mother´s first child. Among the 935 women included in these analyses, 317 (33.9%) remained within a BMI<25kg/m^2^ (R-MBI) and 478 (51.1%) remained OWO (≥25kg/m2) throughout the study period. Maternal demographic characteristics at time of recruitment by *Post-partum BMI pattern* are displayed in Table 1. The percentage of women in the R-BMI group decreased as parity increased from 43.26% to 30.22% (p<0.001). Even that there was a significant difference in schooling with respect to *Post-partum BMI pattern* (p = 0.048), it is not meaningful. Women attended on average to three study visits during the first year post-partum and 82.78% attended to all of the visits designed for its corresponding cohort.

**Table 1:** Descriptive characteristics of the study population by Post-partum BMI pattern.

		Post-partum BMI pattern				
Maternal Characteristics	N = 935	1: Remained with R-BMI	2: Remained overweight or obese	3: Moved from overweight or obese to R-BMI	4: Moved from Normal to overweight or obesity BMI	p-value*
		N = 317 (33.9%)	N = 478 (51.1%)	N = 82 (8.8%)	N = 58 (6.2%)	
**Age at delivery (years)**	26.21	24.64	27.31	26.54	25.33	<0.001
**Education (years)**	10.39	10.10	10.61	10.61	9.83	0.048
**With partner (%)**	90.56	90.54	91.16	93.90	81.03	0.060
**Parity (%) (n = 804)**						
Primiparous	26.74	43.26	40.00	10.23	6.51	<0.001
Multiparous (2,3 and≥4)	73.26	30.22	55.18	8.49	6.11	
**Children characteristics**						
**Male (%)**	51.34	34.38	50.21	9.17	6.25	0.939
**Birth weight (kg)**	3.13	3.07	3.17	3.16	3.08	0.007
**Birth length (cm)**	50.10	49.84	50.21	50.22	50.36	0.058
**Premature (%)**	7.20	8.25	6.93	3.70	8.62	0.001
**Breast feeding duration (months)**	8.13	8.33	8.10	8.14	7.29	0.735
**Overweight children at different ages (moa)**	**% (n)**	**% (n)**	**% (n)**	**% (n)**	**% (n)**	
12 (n = 704)	4.83(34)	5.53(13)	4.34(16)	1.59(1)	10.81(4)	<0.001
18 (n = 683)	6.30(43)	3.45 (8)	9.14(32)	0.00(0)	7.89(3)	<0.001
24 (n = 713)	7.01(50)	4.20(10)	9.12(33)	6.15(4)	6.25(3)	<0.001
30 (n = 535)	7.85(42)	6.10(10)	10.03(29)	1.89(1)	6.90(2)	<0.001
36 (n = 625)	8.00(50)	3.96 (8)	11.18(36)	8.93(5)	2.22(1)	<0.001
42 (n = 228)	3.51 (8)	2.02 (2)	6.45 (6)	0.00(0)	0.00(0)	<0.001
48 (n = 604)	7.45(45)	5.80(12)	9.32(29)	3.77(2)	6.06(2)	<0.001
60 (n = 302)	10.93(33)	7.32 (6)	14.20(24)	5.41(2)	7.14(1)	<0.001
**Weight for height z-score at different ages (moa)**	**μ(n)**	**μ(n)**	**μ(n)**	**μ(n)**	**μ(n)**	
12	0.18(704)	0.13(235)	0.20(369)	0.12(63)	0.39(37)	0.809
18	0.34(683)	0.20(232)	0.47(250)	0.28(63)	0.19(38)	0.093
24	0.31(713)	0.16(238)	0.44(362)	0.27(65)	0.21(48)	0.073
30	0.38(535)	0.10(164)	0.56(289)	0.40(53)	0.15(29)	<0.001
36	0.29(625)	0.05(202)	0.47(322)	0.44(56)	-0.03(45)	<0.001
42	0.21(228)	-0.09 (99)	0.52 (93)	0.40(15)	0.14(21)	<0.001
48	0.45(604)	0.30(207)	0.60(311)	0.39(53)	0.02(33)	0.001
60	0.43(302)	0.18 (82)	0.56(169)	0.46(37)	0.23(14)	0.155
**Average**	0.32	0.14	0.46	0.33	0.12	<0.001

Characteristics of the offspring by *Post-partum BMI pattern* are displayed in Table 1. Overall, 51.3% of the children were males with a mean of weight and length at birth of 3.13(0.42) kg (95% CI: 3.10, 3.16) and 50.1(2.3) cm (95% CI: 49.9, 50.2), respectively. Seven percent were premature. On average, children were breastfed 8±6 months with no difference between *Post-partum BMI pattern*.

On average, children attended 5.3 visits, out of the 7 or 8 scheduled according to the specific research protocol, 27.6% attended all visits designed in its corresponding cohort (data not shown in tables). Overweight children percentage tends to be higher among the OWO mothers all along from 18 to 60 moa. The prevalence of obese infants at 60 months in group from OWO mothers was 14.20% vs 7.32% and in the group from R-BMI. The overweight percentage difference goes up to 7.2% at 36 moa between OWO group (11.18%) and recommended BMI group (3.96%).

**Fig 1:**
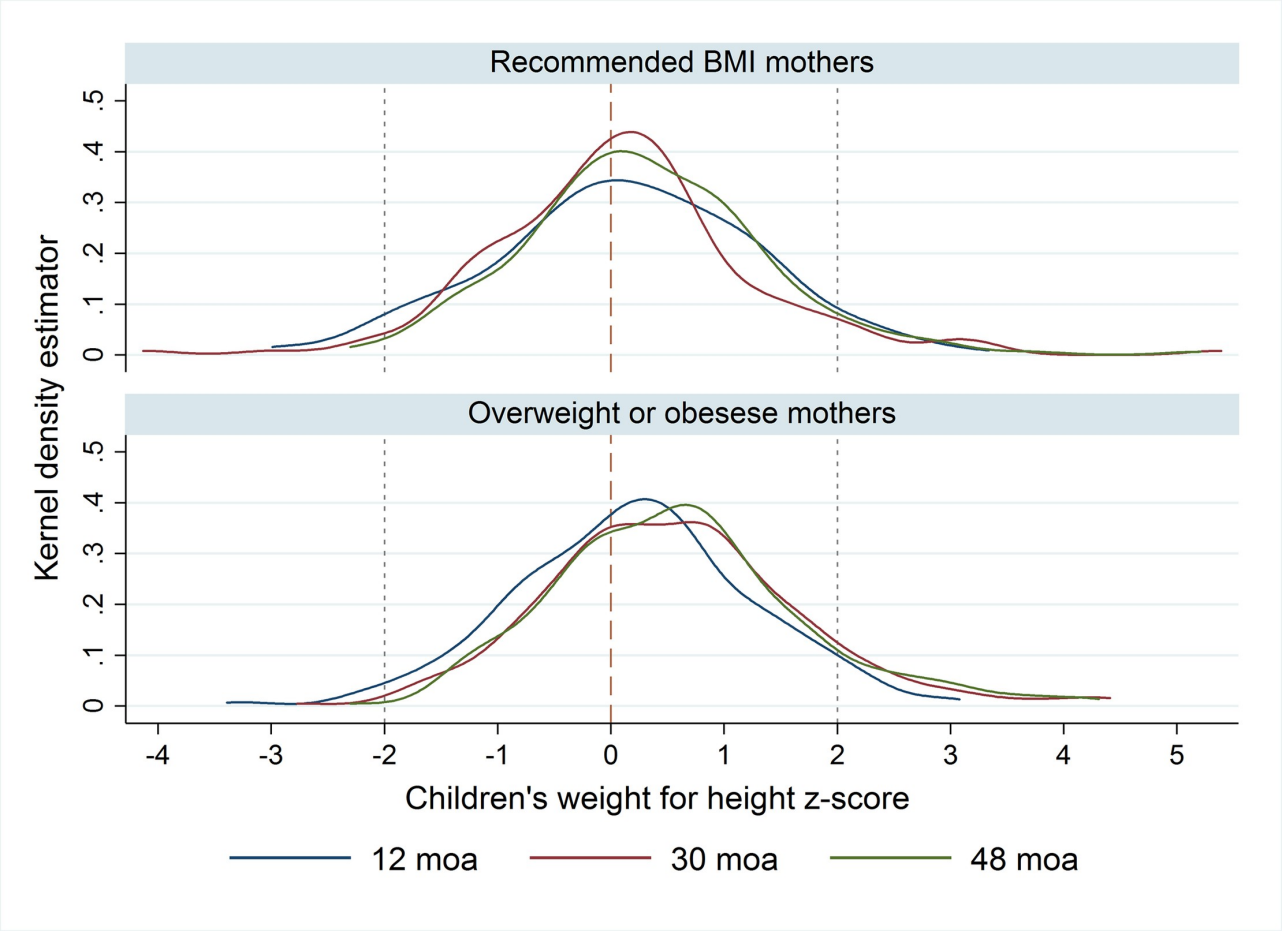
Kernel density estimator of children’s weight for height Z-score by Post-partum BMI pattern.

Children from mothers that remained in the R-BMI group had lower WHZ along all study visits, in comparison with those from women that remained in the OWO category (Table 1). The highest difference was found at 42 months of age, when children’s WHZ was, on average, 0.61 SD different between women that remained with recommended BMI in comparison with those that remained in the OWO category. This positive difference is not limited to mean values; it was systematically found along the whole distribution. Fig 1 shows the distribution of children´s WHZ within the two groups of mothers at three different ages where it can be seen that density functions for the category of OWO mothers are right-shifted with respect to the group of mothers in R-BMI group. Similar behavior was found at any other children age.

### Children’s caloric intake

**Fig 2:**
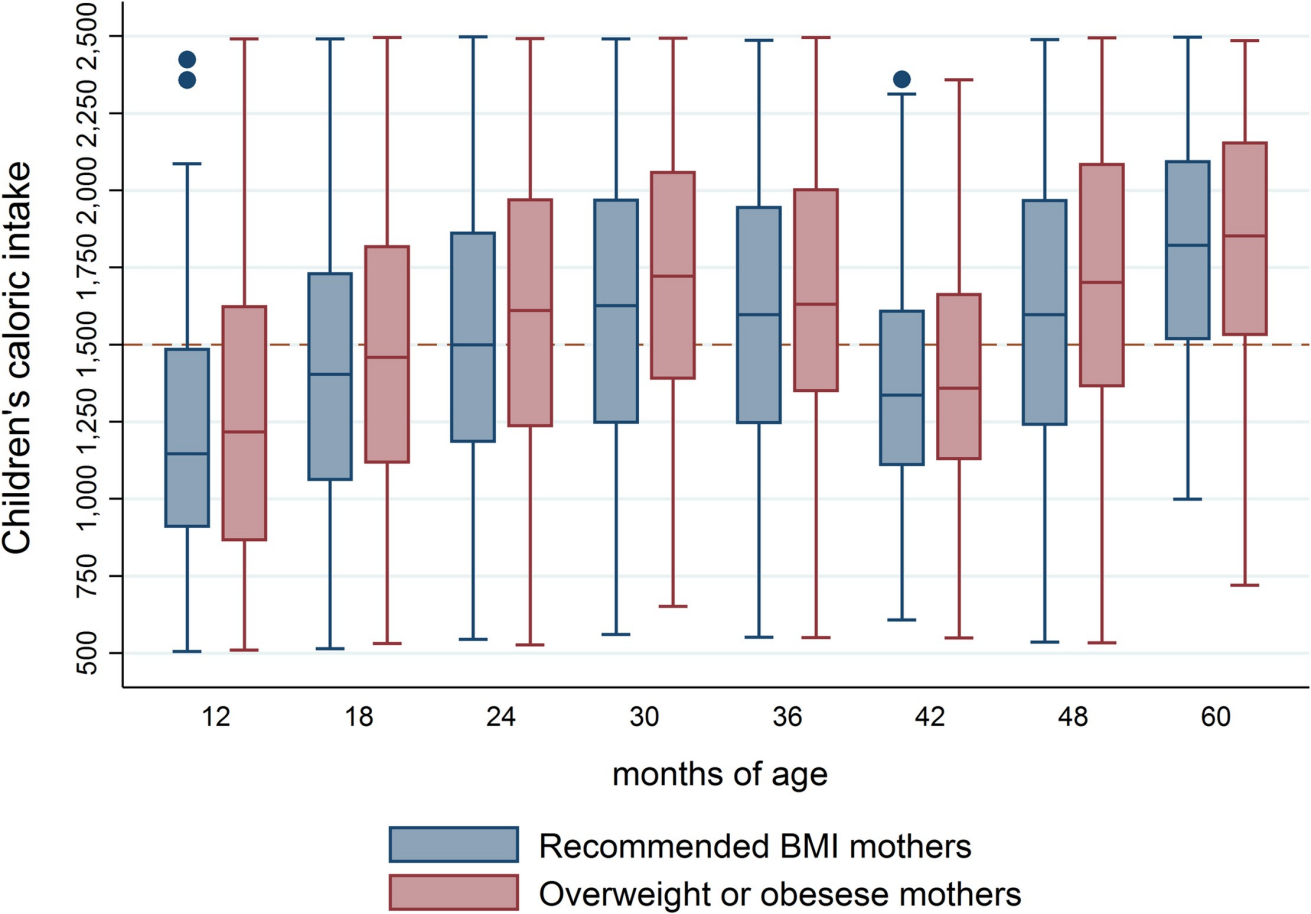
Children’s caloric intake along the study period by PP-BMIP.

The children’s caloric intake increased over time. For each age, the distribution of caloric intake behaves as a mixture of distributions with a bimodality; this was confirmed by the adjustment of a 2-normal mixture model. Children’s caloric intake in the OWO group is consistently higher than the corresponding to the R-BMI mothers (Fig 2). Left panel of Table 2 shows the parameters estimators of each mixture. At 12 months of age, mothers reported almost a 700 kcal difference in children’s intake between these two components or groups (849 vs 1557 kcal). This difference was statistically significant and remained or even increased in the following ages, suggesting that the study population is integrated by two subgroups of infants: those with a *recommended caloric intake* (RCI) and those with a *high caloric intake* (HCI). The proportion of children in the first category globally and by *Post-partum BMI pattern* is displayed in the right panel of Table 2.

**Table 2:** Mixed distribution of the infant’s caloric intake group.

			Component 1: Recommended caloric intake (RCI)		Component 2: Over caloric intake (HCI)		Percentages associated to the second component (HCI)				
							Post-partum BMI pattern				
Months of age	Mean	n	Mean	SD	Mean	SD	HCI	1: Remained with R-BMI	2: Remained overweight or obese	3: Moved from overweight or obese to R-BMI	4: Moved from Normal to overweight or obesity BMI
							n	%	%	%	%
**12**	1231	704	849	166.2	1557	317.7	380	32.9	54.5	7.6	5.0
**18**	1460	683	996	173.6	1725	313.1	434	32.0	52.1	9.9	6.0
**24**	1562	713	1370	337.8	2177	165.8	169	30.2	55.6	6.5	7.7
**30**	1683	535	1454	317.7	2199	162.5	164	26.2	57.3	10.4	6.1
**36**	1638	625	1442	314.9	2199	147.6	162	30.2	55.6	6.8	7.4
**42**	1391	228	1249	252.4	1982	191.2	44	40.9	45.5	0.0	13.6
**48**	1665	604	1552	380.6	2362	75.3	84	23.8	66.7	8.3	1.2
**60**	1807	302	1637	299.8	2277	120.9	80	27.5	62.5	5.0	5.0

Most of the children were in the HCI group at 24 months and later, whereas the opposite happened at earlier ages. The percentage of children in the HCI group was higher at all ages in the group of mothers that remained in the OWO category. Adjusted association between children caloric intake group and *Post-partum BMI pattern* is shown in Table 3. Chances for a child of having an HCI were 51% (95% CI: 11%, 106%) significantly higher among mothers that remained in the OWO group than those that were in the R-BMI group. There were no significant differences between mothers that moved from one group to the other.

**Table 3:** Longitudinal comparison of children’s caloric intake group by Post-partum BMI pattern adjusted by sex and child´s age.

	Odds ratio	95% CI	p-value
**Post-partum BMI pattern**			
Remained with recommended BMI*	1		
Remained overweight or obese	1.51	(1.11, 2.06)	0.008
Moved from overweight or obese to recommended BMI	0.90	(0.53, 1.52)	0.706
Moved from Normal to overweight or obesity BMI	1.32	(0.72, 2.42)	0.370
**Sex**			
Female*	1.02	(0.78, 1.34)	0.903
**Child´s age**			
12*	1		
18	1.96	(1.50, 2.56)	0.000
24	0.15	(0.11, 0.20)	0.000
30	0.23	(0.17, 0.31)	0.000
36	0.19	(0.14, 0.26)	0.000
42	0.22	(0.14, 0.35)	0.000
48	0.07	(0.05, 0.10)	0.000
60	0.16	(0.11, 0.23)	0.000

*Reference category

### Models

After adjusting for year of birth, years in school and breastfeeding, we documented that, independently on their caloric intake group, children from mothers that remained OWO showed and overall increase of 0.35 (95% CI:0.21, 0.49) in WHZ in comparison with children from mothers that remained within the recommended category of BMI (Table 4). A protective effect of breastfeeding was also documented on mothers that breastfed 12 months or more. The percentage of variances that is not explained by the model is 38.2% which demonstrate that maternal anthropometry and children’s diet do not fully explain children’s obesity over time. It is also important to notice that de HCI has no effect in the adjusted model.

**Table 4:** Longitudinal comparison of weight for height Z-score between Post-partum BMI pattern.

	Coefficient	95% CI	p-value
**Post-partum BMI pattern**			
Remained with recommended BMI*	0		
Remained overweight or obese	0.35	(0.21, 0.49)	< 0.01
Moved from overweight or obese to recommended BMI	0.26	(0.02, 0.50)	0.032
Moved from Normal to overweight or obesity BMI	0.08	(-0.19,0.34)	0.577
**Caloric intake group**			
Recommended caloric intake (RCI)*	0		
High caloric intake (HCI)	-0.03	(-0.08, 0.02)	0.266
**Birth weight**			
Low birth weight*	0		
Normal birth weight	0.34	(0.64, 0.61)	0.015
Macrocosmic	0.57	(0.06, 1.08)	0.030
**Number of years after recruitment started**	-0.11	(-0.19,-0.03)	0.008
**Square number of years after recruitment started**	0.01	(0.00, 0.02)	0.026
**Years of school**	0.07	(0.02, 0.17)	0.117
**Square years of school**	-0.00	(-0.01, 0.00)	0.175
**Breast feeding practices (months)**			
≤ 3 *	0		
4–6	0.12	(-0.06, 0.30)	0.205
7–11	-0.02	(-0.19, 0.16)	0.856
≥ 12	-0.16	(-0.32, -0.05)	0.058
**Maternal age(years)**	-0.01	(-0.20, 0.00)	0.187
**Intercept**	-0.34	(-0.92, 0.24)	0.249

Longitudinal regression model with random effects adjusted by children diet group, birth weight, year of birth, years in school and breastfeeding.

*Reference category

Our concurrent ethnographic study with a subset of the maternal child populations provides context for 38.2% that remains unexplained by our statistical model. Results from the ethnographic study document show that there are environmental factors beyond mothers BMI that yield overweight children. In the last 25 years calorie-dense processed foods have become more plentiful and affordable in Mexico City. Mexico City is more accurately classified as a “food swamp” rather than a “food dessert”. [54] Fruits and vegetables are abundant and affordable, but so are caloric dense processed foods. All the mothers observed ethnographically, provided their children with daily home cooked meals, but also purchased snack foods and sugar sweetened beverages throughout the day. This observations is supported by the sociological literature demonstrating that cheap calorie dense foods are an affordable means to demonstrate care within families with limited financial resources [55,56].

Thus, changes in Mexico’s food landscape have made it easier to provide care through cheap processed foods than ever before. While heavier mothers tend to provide more of both kinds of food, all mothers described how they provided more food than in the past. Our ethnographic observations not only documented shifts in the quantity and quality of study participants diet, but also the fact that transformations in the food landscape effect how people eat independent of their age and pregnancy condition. An abundance of cheap processed foods shape diets from gestation, through infancy and the life course.

## Discussion

Our results documented that, after adjusting for confounders, children from mothers that remained in the OWO category had 0.3(0.06) overall higher WHZ during the first five years of life with the biggest difference at 4 years of age (0.6SD), stage near to the adiposity rebound [57]. This difference would lead to 14.2% of OWO children at 5 years of age (WHZ≥2), vs 7.32% raised by mothers in the recommended BMI category. More than sixty percent of the variation of the children growth trajectories was explained by differences between the individual effect, suggesting that there are other factors, beyond family and nutrition habits, that contributed to childhood obesity and played a role in shaping children’s bodies. We used our ethnographic research component to elucidate other possible level factors.

OWO mothers tended to report high caloric diets to their children compared to mothers with R-BMI, addressing the *direct* causes of obesity related with caloric intake. It has been documented the stronger relationship between maternal anthropometry and children’s feeding practices [58] and this was confirmed by our ethnographic observations. In Mexico, mothers are mainly responsible for children’s diet and care in early stages of life. Even that there was a statistical significant difference in schooling with respect to *Post-partum BMI pattern* (p = 0.048), we don’t think that education would be related to the way mothers behave in the different groups given that the observed differences are not meaningful. We interpreted maternal anthropometry first as a proxy of the intrauterine environment where over caloric maternal diet is associated to obesity of the offspring [18,20], consistent with previous studies that demonstrated a positive and significant correlation between gestational weight gain and post-partum weight change, showing an increase of almost half kilogram of weight retention per every kilogram increase during pregnancy [47]. Second, as a reflection of an obesogenic environment that promoted the provision of over caloric diets to their children.

Nevertheless, we do not mean to imply that mothers are the only influence on children’s weight. Based on Naess [16], having both parents OWO, doubles the chances of the offspring of being OWO as they are the underlying and basic causes of obesity discussed before for our study population context. Unfortunately, ELEMENT does not have information on father’s anthropometry.

It has been documented that in the first six months of life, birthweight and breast-feeding were the most important determinants in weight gain [54]. This result was confirmed in our sample population. Those children that were breastfed for at least one year showed 0.15 SD less WHZ (p = 0.07) with respect to those breastfeed less than a year.

Our results are in accordance with previous findings in Mexican and Mexican-American [59] population documenting that having an obese mother was significantly associated with being OWO. Previous evidence shows heterogeneous results in childhood, ages 2–7 [60] and a stronger relationship after 10 years [27], with well-established associations in adolescence and adulthood [61]. Longitudinal studies show that the association established in the childhood remains until adulthood [62]^.^

The largest strength of the study is its longitudinal design in both generations, and the complementary strategies of analyses. Complementing statistical analysis from a cohort study with ethnographic observations allowed us to discuss further the environmental and developmental origins of obesity in a comprehensive approach including immediate, underlying and structural factors under Rivera’s framework. However, we also identify some limitations: the study population participating in the ELEMENT cohort studies, is a non-probabilistic sample that was recruited in public hospitals with no population representativeness. In the beginning, the main focus of ELEMENT was towards biological mechanisms where this kind of representativeness is not crucial. However, in this research, we acknowledged that the social environment plays a relevant role, and the socioeconomic homogeneity of the sample may constitute a limitation preventing the extrapolation of the results to a different population where the social environment differs. However, the percentage of the Mexican population that receives health services from the public hospitals is so high (72.58% in 2012 and 83.8% in 2016) [4,63] that we think that our results could be generalized to a large proportion of the Mexico City population. As we mentioned before, we lack on father’s anthropometry, derived from the cohort design.

We neither have quantitative information on children physical activity and on physical environments, but through our ethnographic study, we know that ELEMENT participants live in environments with very limited access to physical activity regularly: The only regular physical activity was walking to school [64]. Finally, we used FFQ to calculate nutrient intakes, where the memory of the respondents plays a major role in their responses [65,66]. Diet evaluation is complex in infants and may entail measurement errors given that: dietary habits change rapidly at that age, parents share their feeding responsibility with other adults (caregivers), children do not necessarily consume all the food they are given [67] and/or mothers under report unhealthy foods.

As these studies highlight the importance of early factors that influences the development of obesity in children, future directions of this research should focus on replicating this analysis with a longer-time effect (adolescence or young adulthood) as this is an ongoing cohort. There needs to be further studies utilizing data from birth-cohorts conducted in developing countries to confirm our findings.

## Conclusions

Maternal obesity during the first year post-partum might reflect an obesogenic environment during gestation provided by an over caloric maternal diet. The obesity condition almost doubled the prevalence of overweight and obesity at five years of age in the offspring with respect to non-obese mothers.

## Supporting information

S1 Table Energy recommendation for mexican population and Infant’s caloric intake group [68]. (DOCX)

## Abbreviations

OWO: Overweight and obesity
moa: months of age
yoa: years of age
ENSANUT: National Survey of Health and Nutrition
BMI: Body Mass Index
PP-BMIP: Post-partum BMI pattern
RCI: Recommended caloric intake group
HCI: High caloric intake group
WHZ: weight for height z-score
R-BMI: recommended BMI group
SES: socioeconomic status
