# Supplementary material for: Influence of post-partum BMI change on childhood obesity and energy intake
Source: PLoS One. 2019 Dec 12;14(12):e0224830. doi: 10.1371/journal.pone.0224830 (PMC6908440; doi:10.1371/journal.pone.0224830)
Supplement: S1 Table — (DOCX) [file pone.0224830.s001.docx]

Supporting information

**S1 Table. This is the S1 Table I Energy Recommendation for Mexican Population and Infant’s caloric intake group.**

|  | **Infant’s caloric intake group** | | | | | |
| --- | --- | --- | --- | --- | --- | --- |
|  | Energy Recommendation for Mexican Population* | | Component 1: Recommended caloric intake (RCI) | | Component 2: High caloric intake (HCI) | |
| **Age** | Girls | Boys | Girls | Boys | Girls | Boys |
| **(months)** | (kcal/d) | (kcal/d) | (kcal/d) | (kcal/d) | (kcal/d) | (kcal/d) |
| **12-23** | 865 | 948 | 895.5 | 929.6 | 1649.4 | 1644.9 |
| **24-35** | 1047 | 1129 | 1412.7 | 1396.4 | 2180.0 | 2196.0 |
| **36-47** | 1156 | 1252 | 1400.5 | 1375.6 | 2156.7 | 2148.8 |
| **48-59** | 1241 | 1360 | 1553.5 | 1552.1 | 2368.0 | 2355.8 |
| **60-71** | 1331 | 1467 | 1650.1 | 1622.8 | 2277.5 | 2277.8 |
| Bourges R. H, Casanueva E, Rosado JL. Recomendaciones de ingestión de nutrimentos para la población mexicana. Editorial Medica panamericana; 2005. | | | | | | |
